# Social Attention and Emotional Responsiveness in Young Adults With Autism

**DOI:** 10.3389/fpsyt.2019.00426

**Published:** 2019-06-19

**Authors:** Renee Dijkhuis, Emine Gurbuz, Tim Ziermans, Wouter Staal, Hanna Swaab

**Affiliations:** ^1^Department of Clinical Child and Adolescent Studies, Neuropedagogics and Developmental Disorders, Leiden University, Leiden, Netherlands; ^2^Leiden Institute for Brain and Cognition, Leiden, Netherlands; ^3^Karakter Child and Adolescent Psychiatry University Center, Nijmegen, Netherlands; ^4^Department of Psychiatry, Radboud University Medical Center, Nijmegen, Netherlands

**Keywords:** autism spectrum disorder, adulthood, social attention, emotional arousal, skin conductance level, symptom severity

## Abstract

Children with autism spectrum disorder (ASD) are generally characterized by marked impairments in processing of social emotional information, but less is known about emotion processing in adults with the disorder. This study aimed to address this by collecting data on social attention (eye tracking), emotional arousal (skin conductance level, SCL), and emotional awareness (self-report) in a paradigm with social emotional video clips. Fifty-two young, intelligent adults with ASD (IQ_range_ = 88–130, Age_range_ = 18–24) and 31 typically developing (TD) ASD (IQ_range_ = 94–139, Age_range_ = 19–28) gender matched controls participated and reported on severity of autism symptoms [Social Responsiveness Scale for Adults (SRS-A)]. Results showed no group difference in social attention, while autism symptom severity was related to decreased attention to faces across participants (*r* = −.32). Average SCL was lower in the ASD group, but no group difference in arousal reactivity (change from baseline to emotional phases) was detected. Lower SCL during video clips was related to autism symptom severity across participants (*r* = −.29). ASD individuals reported lower emotional awareness. We conclude that, even though no deviations in social attention or emotional reactivity were found in ASD, an overall lower level of social attention and arousal may help explain difficulties in social functioning in ASD.

## Introduction

Autism spectrum disorder (ASD) is a complex condition with impairments in social communication and behavior. In the search for mechanisms that underlie the disorder, it is well established that children with ASD show deviant emotional processing, as is reflected in lower arousal levels when processing emotions of others (1, 2) and difficulty in recognition of facial emotions in others (3). Atypical attention towards faces and social sensitivity in ASD persists into adulthood (4, 5) and is also evident in ASD without associated intellectual disability, particularly when more sensitive measures are used (6). Behavioral investigation of social emotional processing showed that autistic individuals are slower to make emotional attributions even though their emotional attributions were accurate (7). Neural investigation of emotional attributions to body language showed less coherent brain activation in children with ASD compared to their neurotypical peers (8). Studies looking at self-reports of individuals with ASD have found that they are less able to recognize and identify their own emotions (9, 10) and emotions of others (1), and it has been argued that such socio-cognitive abnormalities are related to symptom severity (11, 12). However, these neuropsychological and physiological mechanisms might be differently associated to symptom severity at different ages since they are still maturing in children and studies focusing on mechanisms underlying social emotional functioning in adults with ASD are scarce. Furthermore, most studies focus on how individuals with ASD attend to and interpret the emotions of others while less is known about how they process information in social emotional contexts themselves. This study aims to look into social attention and emotional responsiveness in young adults with ASD.

Changes in cognitive and emotional states are reflected in activity of the peripheral autonomic nervous system (ANS). Skin conductance (electrodermal activity) is often used as an implicit measure of attention, cognitive effort, or arousal (13, 14). Electrodermal responses are produced for example when individuals are presented with emotional facial expressions (15), and it has been suggested that this arousal modulates emotional processing, social cognition, and motivational decision-making (16). Previous research in ASD populations has shown mixed results in terms of arousal reflected in skin conductance level (SCL). One study reported significantly lower baseline arousal in children with ASD (17), while another reported no group differences compared to typically developing (TD) children and adolescents (18). In response to social emotional video clips, typical arousal has been demonstrated in adolescents and adults with ASD (19). Trimmer et al. (19) also measured self-reported arousal response and found that the ASD group did not differ from the control group in their awareness of perceived arousal following the emotional clips. Although seldom included alongside more subjective measures of emotional arousal, self-awareness of internal emotional experiences is considered a fundamental prerequisite for adequate coping with the resulting emotional consequences and for managing associated behavioral impulses. The results found by Trimmer et al. (19) are in line with an earlier study by Dziobek et al. (20). Dziobek and colleagues found that the ASD adults had no difficulties in rating their own emotional reactions in response to emotional photos, compared to controls. However, Bölte et al. (21) reported lower awareness of arousal in adults with autism when viewing sad stimuli compared to TD controls, which was not reflected in their heart rate, suggesting deviant experience of emotional arousal in ASD. Additionally, autism symptom severity has been associated with greater skin conductance responses (SCRs) to nonsocial than to social stimuli (22). The authors suggested that the failure to orient to socially salient information could be framed as reduced motivation (resulting from lower levels of arousal) for social information in ASD, which is consistent with other studies finding impaired motivation for social situations in ASD (23). Moreover, it has been found that lower baseline SCLs predict impaired emotion recognition and affective empathy in adults with ASD, indicating a relationship between low baseline resting states and altered emotion regulation (24).

Previous studies into facial processing and emotion recognition in ASD have demonstrated that the magnitude of group differences in processing social cues is partially determined by the nature of the task stimuli. Individuals with ASD perform relatively well compared to controls in tasks that use static social stimuli (25, 26). However, when more dynamic social stimuli are used, i.e., with greater resemblance to real-life interactions, individuals with ASD focus significantly more on the mouth, body, and objects compared to controls, and significantly less on the eye region (27). In addition to the dynamic versus static distinction, the social complexity of the stimuli is also important. For instance, Speer et al. (28) examined four different conditions in children with ASD: static-nonsocial, static-social, dynamic-nonsocial, and dynamic-social. Children with ASD looked less at the eyes and more at the body of the characters compared to their TD peers in the social dynamic condition, while no significant group differences were found in any of the other conditions. These findings suggest that processing of social emotional cues is partially determined by the realistic nature of the stimuli. Moreover, it was found that fewer fixations on the eyes in the ASD group were correlated with severity of autism symptoms. In a review by Chita-Tegmark (29) it is concluded that social content (defined as “low” if there is one person presented and “high” if two or more people are presented) is the most significant predictor of social attention in ASD and that differences with TD individuals are larger when the social stimuli are more complex (e.g., more than one person).

The first aim of the current study is to investigate whether social attention and emotional responsiveness in young adults with ASD differ from their TD peers. The second aim of the study is to investigate whether social attention and emotional arousal relate to autism symptom severity. To address these aims, we measure social attention and emotional arousal during dynamic social situations. We hypothesize that young adults with ASD attend less to essential social cues (faces) and display reduced arousal and reactivity (change in arousal) in response to emotional social situations. Based on earlier studies we also expect social attention and emotional arousal to be associated with severity of autism symptoms. As a final aim, we are interested in assessing emotional awareness as part of emotion regulation in ASD. Emotional awareness is expected to be lower in the young adults with ASD compared to their TD peers.

## Materials and Methods

### Participants

Fifty-one young adults with ASD (*M_age_* = 22.46, *SD* = 2.52; 72.5% male) and 27 TD individuals (*M_age_* = 20.65, *SD* = 1.57; 74.1% male) participated in the present study. All participants were postsecondary students enrolled in university programs or at universities of higher professional education in the Netherlands. Both males (*n* = 57) and females (*n* = 21) were included in the study and the groups were matched on gender. The ASD group was recruited through Stumass: a Dutch non-profit organization providing assisted living services for higher education students with ASD. In order to be enrolled into Stumass, applicants are required to have received a formal clinical diagnosis of ASD based on the *Diagnostic and Statistical Manual of Mental Disorders* (DSM) customary at the time of referral (DSM-III-R/DSM-IV/or DSM-IV-TR), according to protocolled guidelines in the Dutch mental health system. An additional requirement for enrollment in Stumass is that psychiatric co-morbidity, if present, is either in remission or under supervision of a certified psychologist or psychiatrist. For the control group, higher education students from the city of Leiden and neighboring regions were recruited through information brochures and an online student recruitment platform at Leiden University. Controls who reported having received a DSM diagnosis during their lifetimes were not included in the study. An additional inclusion criterion for both groups was an intelligence quotient (IQ) of 80 or higher, which was checked with the two subtests Vocabulary and Block design of the Dutch version of the Wechsler Adult Intelligence Scale–Fourth Edition (WAIS–IV) (30), known as the V-BD short form. Total IQ was estimated based on a long-standing method in the short-form literature with the formula [3 × (sum of normed scores) + 40] (31). For their participation in the study, all participants were rewarded with a voucher of 20 euros and a written summary of their cognitive strengths and difficulties in the study. The research protocol was approved by the Medical Ethics Committee of Leiden University Medical Center. In accordance with the Declaration of Helsinki, written informed consent was obtained from all participants before participation.

### Measures

#### Autism Symptom Severity

Severity of autism symptoms was measured with the Dutch self-report version of the Social Responsiveness Scale for Adults (SRS-A) (32). The SRS consists of 65 questions in which higher scores indicate more social impairment and more severe ASD traits. Internal consistency was found to be highly acceptable in a German cohort with Cronbach’s alpha ranging from 0.71 (TD participants) to 0.89 (autism participants) (33), and the overall test–retest reliability (Pearson’s *r*) for the SRS-A was found to be 0.64 (32).

#### Complex Dynamic Social Video Clips

**Figure 1:**
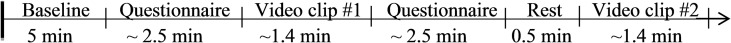
The social emotional paradigm; followed up by video clips #2, #3, and #4 with accompanying questionnaire and rest.

Social attention and emotional responsivity of the participants were assessed during a social emotional paradigm (SEP), which included video clips of real-life social situations with high emotional content. Ten publicly broadcasted videos including humans in real-life social interactions with a high level of emotional content were selected from the Internet. These videos were piloted with 10 university students without ASD. Appropriateness of the stimuli was determined by analysis of a) average SCL and b) congruence between self-reported emotional awareness (as indicated by type and intensity of a pre-selected set of emotions) and content of the video clips (e.g., high intensity of happy emotions during a sad clip was considered incongruent). As a result, the four video clips that elicited the highest reactivity (arousal difference between baseline and stimuli) were chosen to be used in the actual study. These video clips contained auditory input (e.g., speech, scream, and cheers) and varying emotional content. Spoken language in the video clips was English. The description of each video clip and the emotions they include are presented in **Table 1**. In addition, one neutral video of an aquarium with sound was presented for 5 min as a baseline measure, which has been shown to be an adequate measure of resting state (34). Social emotional video clips were presented in a counterbalanced fashion, and each clip lasted 75 s on average. Between each social emotional video clip, 30 s of a neutral video clip showing an aquarium was presented in order to prevent arousal from accumulation with time. A visual representation of the paradigm timeline is displayed in **Figure 1**.

**Table 1:** Description of social emotional video clips.

	Description	Emotion
Video clip 1	A young woman surrounded by family is in suspense at the airport to reunite with her boyfriend. When they see each other, they are overcome with joy and hug intensely.	Happy/surprise
Video clip 2	A man and a woman surrounded by an angry crowd are in a heated discussion at a public demonstration.	Anger/irritation
Video clip 3	A close-up of a heartbroken former athlete on a podium who publicly announces the death of an American football legend in a stadium	Sad/sorrow
Video clip 4	A close-up of the face of a woman on a dental chair who is undergoing a nipple-piercing procedure in a body art shop	Pain/fear

#### Social Attention

The video clips were displayed on a 15.6-in. Liquid crystal display (LCD) screen, and gaze data were collected by a Tobii T120 Eye Tracker (Tobii Stockholm, Sweden). Gaze fixations and pupil responses were sampled at a frequency of 120 Hz. An Identification by Velocity Threshold (I-VT) fixation filter was applied to the data collected from both eyes. The face areas of interest (AOIs) included eyes and mouth and were drawn sufficiently large outside of the defining contours to reliably capture the gaze fixations (35). A fixation was registered if the velocity threshold for an eye movement exceeded 30°/s within a 40-pixel diameter region (36). Each participant’s gaze data for both eyes were checked in Tobii Studio to remove any outlier values due to blinks, loss of tracking data, and small sampling size, or large moves in head position. Five dynamic AOIs were defined for each video clip: face, eye, mouth, whole screen, and background (whole screen excluding face AOIs). The total visit duration (in seconds) at the whole screen was computed for all video clips in order to control for overall attention to the stimuli presented and total fixation duration at each AOI was calculated to measure attention towards social (e.g., face, eyes) and nonsocial stimuli (e.g., background). Fixation ratios (% fixation at AOI relative to the visit duration at overall screen) were computed for each AOI. The visit duration at the overall screen and fixation ratios at the AOIs in all four video clips were summed to be used as dependent variables in the analysis.

#### Emotional Arousal

Throughout the experiment, electrodermal activity measurement was acquired through a galvanic skin response amplifier (EDA100C) by Biopac data acquisition system (Bionomadix MP150-Windows). Two contact Ag/AgCl disposable electrodes (Biopac EL507) were attached to the middle phalanges of the second and fourth fingers of the participants’ non-dominant hand. The EDA data were collected with a rate of 1,000 samples/s in standard units of microSiemens (μS) using the *Acqknowledge* software (Biopac System Inc). The emotional arousal data were synchronized with the eye tracker by manually entering event markers to indicate the start and the end of each video clip. In *Acqknowledge*, a 0.05-Hz high-pass filter was applied to create a phasic channel from the tonic channel. The low-pass filter was set to 1 Hz in order to filter high-frequency artifacts from the raw signal. Recorded data were further processed by manually inspecting the SCL using the *Acqknowledge* software. Movement artifacts were visually identified and excluded from the data. An analysis script in Matlab Release 2016a (The MathWorks, Inc., Natick, MA, USA) was used to automatically analyze the data. The mean SCL (μS) was calculated per minute of the baseline and for all video clips. The lowest SCL during 1 min of the baseline was chosen for each participant to be able to control for individual differences in baseline SCL in further analysis.

#### Emotional Awareness

After each clip, participants were asked to describe what happened in the video clip by digital query on a tablet, to make sure they fully understood the content of the video. Participants were also asked to indicate if they felt emotions themselves and to what degree as indicated by a set of nine different emotions (angry, upset, irritated, nervous, happy, surprised, optimistic, sympathetic, and horrible) on a continuous line of 10 cm with a scale from 0 to 100 for each of the emotions. The specific emotions were derived from Parrott (37) and a similar questionnaire has been used before, for example by van Rijn et al. (38).

### Procedure

The information letter about the study was sent by mail and participants were included after they returned their written consent. Control participants were tested at Leiden University and ASD participants were tested in Stumass residential homes. In all cases, the experiments were conducted in a quiet and stimulus-free room during daytime. There were two parts of the experiment; the first part was the administration of the WAIS by a trained psychology student followed by SRS-A completed by the participant. The second part was the experimental part where the psychophysiology and eye-tracking measures were collected. After the electrodes were attached, participants were instructed to put their hand on the table in a resting position without moving or touching the electrodes. The participants were placed in front of the Tobii monitor, with a 60-cm distance from the screen. A nine-point calibration of the eye movements was applied, and it was repeated until the participant’s eye gaze could target all of the nine points on the screen. Participants were instructed to look at the screen before the task was started. Task duration was approximately 35 min.

### Statistical Analyses

Statistical tests were conducted in IBM SPSS (v.21). Level of significance was determined at *p* < 0.05, and in the case of relevant group differences, Cohen’s *d* was calculated as a measure of effect size. First, the autism and control group were compared on age, IQ, and autism severity with independent *t*-tests and on gender with a chi-square test. For eye-tracking data, separate one-way ANOVAs were performed to analyze the difference in total visit duration and fixation ratios (face, eye, mouth, and background) between the ASD and the TD group. Additional Pearson correlations were computed between SRS-A total score and total visit duration at the whole screen as well as fixation ratio to the face for all participants.

For the skin conductance data, SCL (μS) during baseline and video clips was checked for normality of the distributions using histogram and boxplots in SPSS. Baseline SCL was compared between the groups using an independent samples *t*-test. Since we were interested in general arousal to the video clips, regardless of the specific emotions or content, the group mean SCL to all video clips (stimuli) taken together was used as a dependent variable. In order to test for group differences in SCL reactivity, a repeated measures analysis of variance (RM-ANOVA) was conducted with condition (baseline; social emotional video clips) as within-subject factor, and group (ASD; TD) as between-subject factor. Additional Pearson correlations were computed between SRS total score, SCL during baseline, and SCL during stimuli for all participants.

We checked for influence of covariates on baseline SCL (total IQ, age, and gender) and all eye-tracking measures (total IQ and age) by applying correlational analysis. No significant correlations were found so it was decided not to control for covariates in the analysis.

For the analyses on emotional awareness, it was first determined for each video clip which two emotions were experienced most intensely in the TD and ASD groups separately. Second, it was tested whether the ASD group experienced different intensity levels on the most salient emotions in the TD comparison group. To achieve this, a multivariate ANOVA with eight emotion ratings in total (2 × 4) was performed to investigate group differences in emotional awareness elicited by the presented video clips.

## Results

The sample characteristics are given in **Table 2**. The groups were matched with regard to sex [*χ* ^2^ (1) = .02, *p* = .55], while the ASD individuals were significantly older than the TD group [*t* (73.79) = 3.91, *p* < .001, *d* = −0.8]. The estimated IQ scores were also different between groups such that ASD participants had higher scores than the TD group [*t* (76) = 3.97, *p* < .001, *d* = −0.9]. The individuals with ASD reported to have significantly higher SRS total scores [*t* (73) = 4.99, *p* < .001, *d* = −1.2] and significantly higher subscale scores compared to controls (*p* < .001). At the time of assessment, 19 participants in the ASD group were on (multiple) prescribed medications, including the stimulant methylphenidate; see **Table 2**. As not much is known about the exact effects of these medications on arousal and existing studies do not clearly point to attenuation or elevation of arousal, we decided to perform SCL analyses both with and without participants that used medication.

**Table 2:** Group characteristics.

	ASD (n = 51)	TD (n = 27)
Male (% in group)	72.5	74.1
Age, in years, *M* (*SD*)	22.46 (2.51)	20.65 (1.57)
WAIS-IV Total IQ, *M* (*SD*)	118.24 (10.75)	107.78 (11.69)
SRS Total Score*, *M* (*SD*) Methylphenidate (*n*)	62.78 (10.11) 6	50.04 (11.24) 0

WAIS-IV, Wechsler Adult Intelligence Scale–Fourth Edition; IQ, intelligence quotient.

*SRS-A t-score; missing data in the ASD group (n = 2) and the control group (n = 1).

### Social Attention

**Figure 2:**
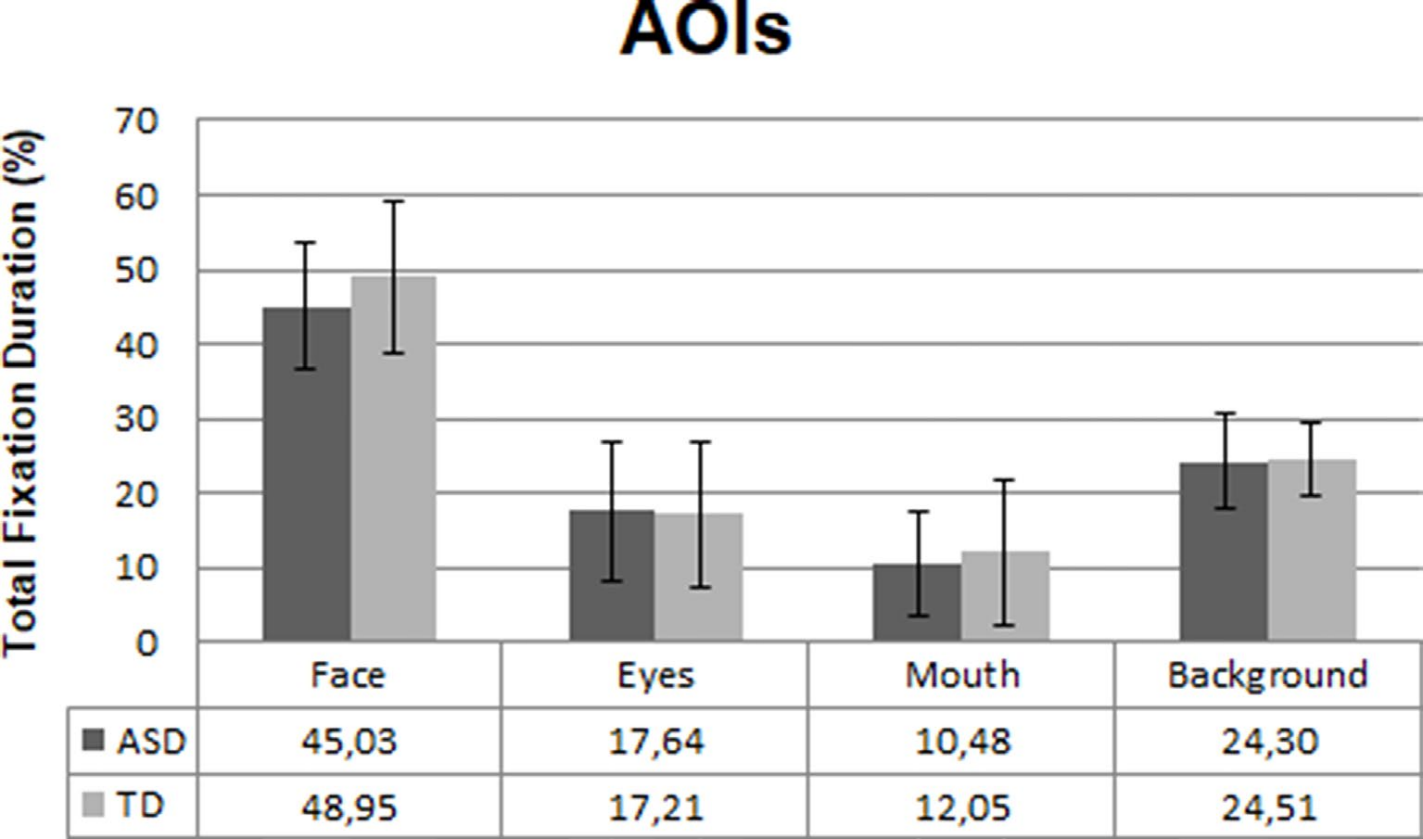
Mean fixation duration in the ASD (*n* = 49) and TD (*n* = 26) group at the four areas of interest (AOIs) in percentages. Error bars represent standard deviation of the mean.

The eye gaze data from three participants (ASD; *n* = 2, TD; *n* = 1) were excluded from the final analysis due to poor calibration and/or overall looking less than 10% at the whole screen. Consequently, the final eye-tracking analysis was conducted with 49 ASD and 26 TD participants. There were no differences between the groups for total gaze duration at the overall screen [*t* (73) = 1.2, *p* = .16, *d* =−0.2], indicating that the groups paid equal attention to the video clips. There was no significant group difference in total fixation duration at the face AOIs in the video clips. However, the ASD individuals showed a trend (*p* = .08, *d* = 0.4) towards looking less at faces than the TD group; see **Figure 2**. No significant differences were found between the two groups in fixating at eyes (*p* = .85), mouth (*p* =. 43), or background (*p* = .89) in the video clips.

**Figure 3:**
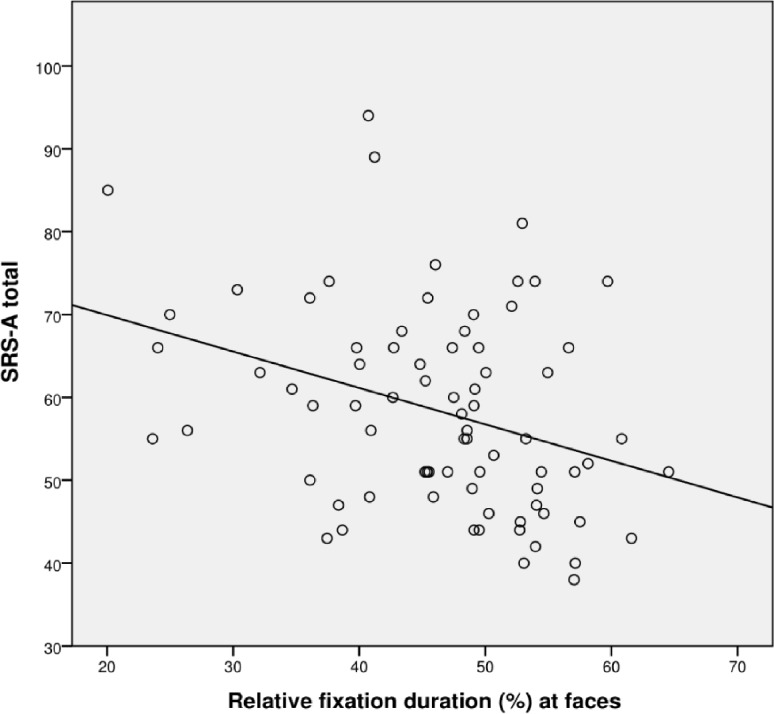
Scatterplot for correlation between autism severity (SRS-A Total) and percentage fixation at faces for all participants (*N* = 72).

The correlation between SRS total score and total visit duration at the whole screen was not significant (*r* = −.161, *p* = .18), while the correlation between SRS total score and relative fixation duration at faces was significant (*r* = −.323, *p* = .01); see **Figure 3**.

### Emotional Arousal

**Figure 4:**
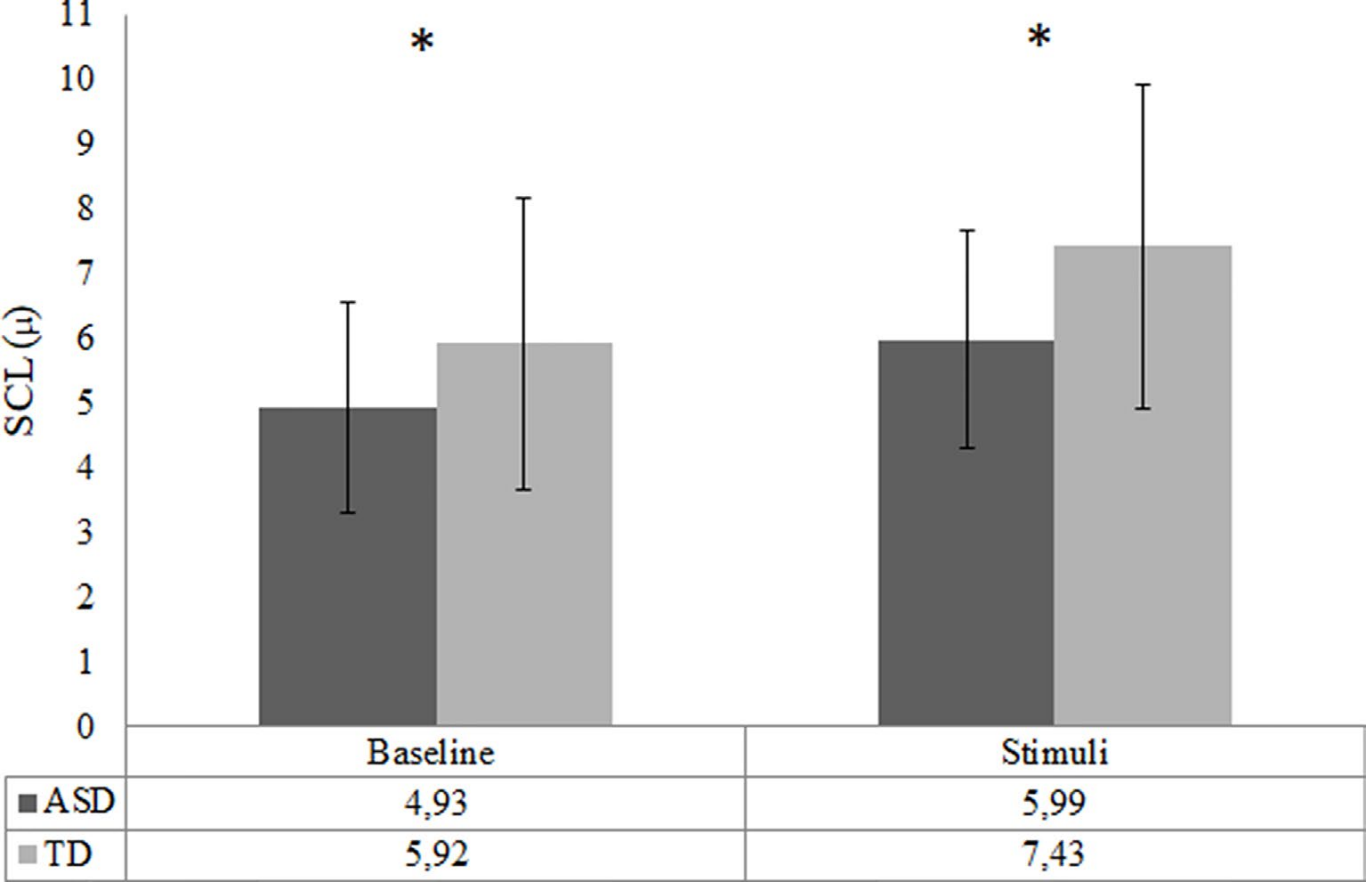
Arousal during baseline and task in the ASD (*n* = 38) and the TD (*n* = 25) group—indicated by skin conductance level (SCL; mean). Error bars represent standard deviation of the mean; *p < .05.

**Figure 5:**
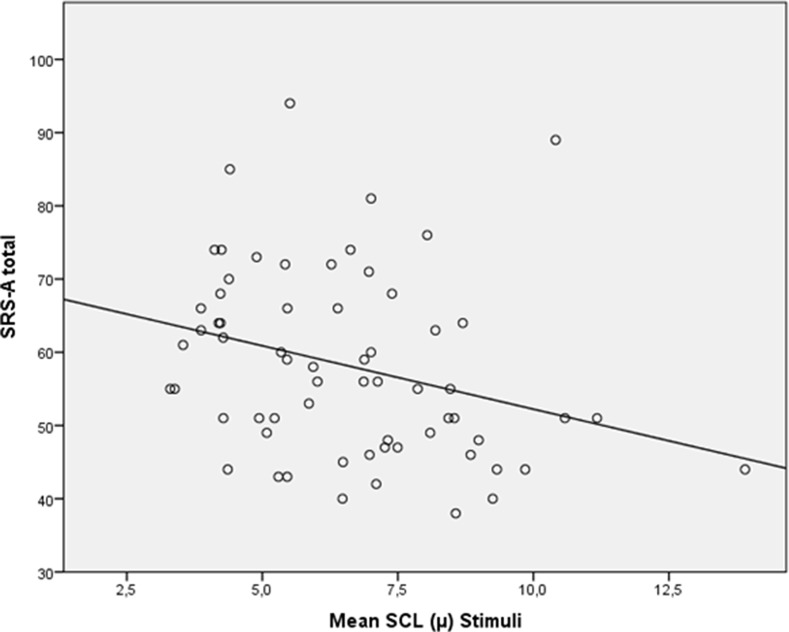
Scatterplot for correlation between autism severity (SRS-A Total) and skin conductance level (SCL; mean) during stimuli for all participants (*N* = 61).

The analysis of EDA did not include two TD participants and 12 ASD participants due to poor quality of data as a result of various technical issues. Consequently, the final SCL analysis was conducted with 38 ASD and 25 TD participants. There was a trend-significant difference in baseline SCL between the ASD and TD group, *t* (40.14) = −1.99, *p* = .05, *d* = 0.5. The baseline SCL in the ASD group was lower compared to the TD group; see **Figure 5**. The RM ANOVA analysis showed a main effect of condition [*F* (1, 61) = 135.10, *p* < .001], for SCL, indicating that all participants had higher arousal during video clips compared to the baseline. The RM ANOVA analysis to test change from baseline SCL to social emotional video clips did not result in any group by condition interaction effect, *p* = .12. However, there was a main effect of group [*F* (1,61) = 9.84, *p* = .003, *d* = 0.8]; ASD participants showed significantly lower SCL responses during video clips compared to TD controls; see **Figure 4**. Repeating the SCL analysis without participants that used methylphenidate did not affect the outcome in terms of significance.

When looking at all participants (*n* = 61), a trend-significant correlation between baseline SCL and SRS total score appeared (*p* = .07), and a significant correlation between SRS and SCL during processing of the social emotional information was shown (*r* = −.350, *p* = .01); see **Figure 5**.

#### Emotional Awareness

**Figure 6:**
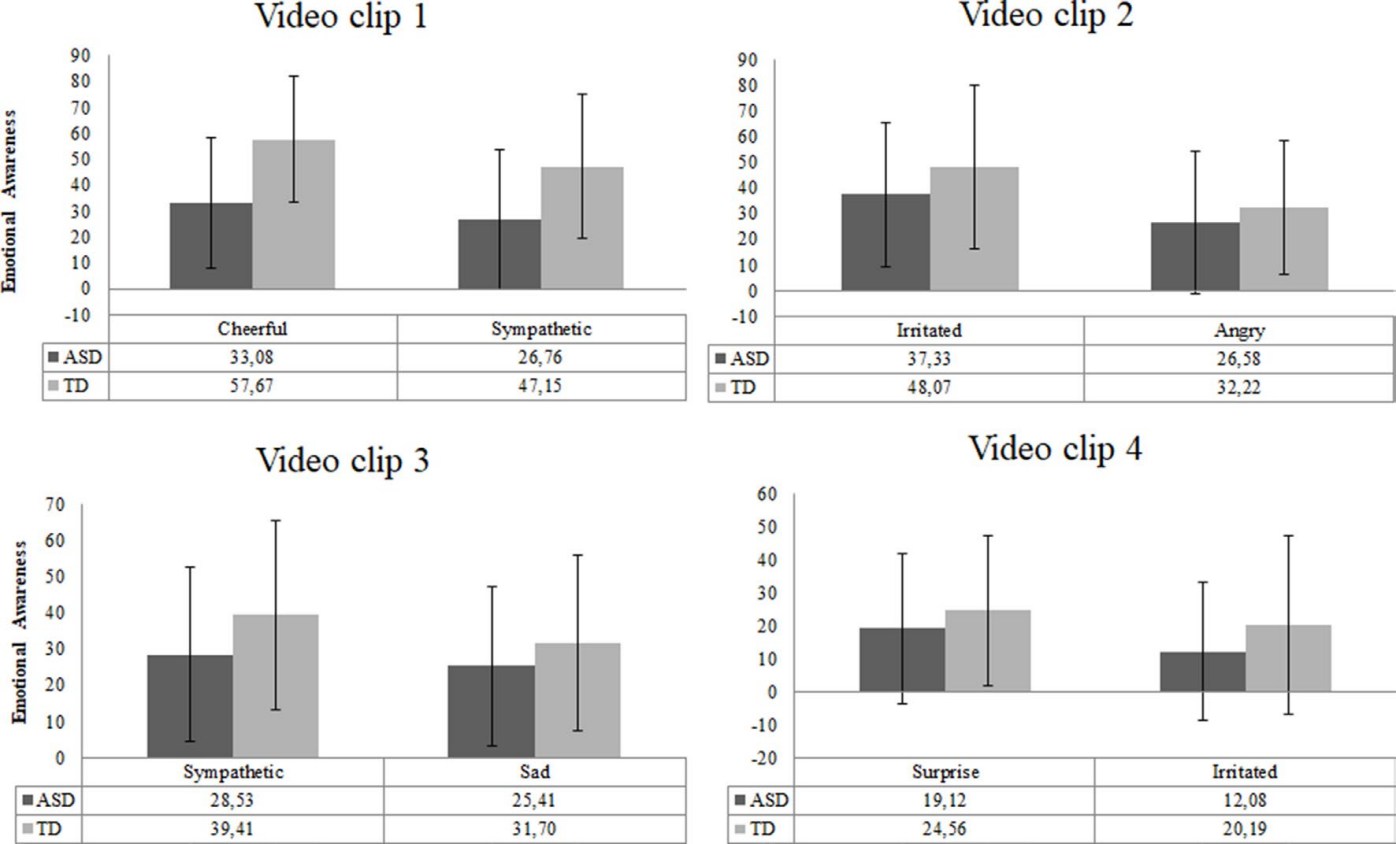
Emotional awareness reported for each video clip in the ASD (*n* = 51) and TD (*n* = 27) group. Error bars represent standard deviation of the mean.

The scores of intensity ratings showed that both TD and ASD participants chose the exact same two emotions for each video clip (**Figure 6**). This result indicated that video clips elicited similar emotions in both groups. However, the overall intensity levels of these emotions were perceived as significantly lower by ASD individuals than by TD individuals [*F* (8,69) = 2.29, *p* = .03, *d* = 0.8].

## Discussion

The primary goal of this study was to investigate social attention and emotional responsiveness to emotional situations in adults with and without ASD. Contrary to our expectations, the results did not show a significant difference in social attention between young adults with ASD and their peers, although there was a trend towards less attention for faces in autism. However, lower baseline arousal and lower arousal during processing of social emotional information was observed in ASD individuals, while reactivity from baseline to social emotional stimuli did not differ between the groups. The second goal of the study was to investigate whether social attention and emotional arousal can be related to autism symptom severity. Lower fixation on faces indicated higher severity of autism symptoms. Also, higher levels of arousal were found to be associated with lower symptom severity. The association between both social attention and emotional arousal with autism symptom severity suggests that they could be candidates for broader phenotype autism traits (39). Finally, in line with our expectations, ASD participants reported lower intensity of emotional awareness when exposed to social emotional stimuli compared to their TD peers.

The finding of no differences in social attention between the autism and the control group in this study is not in line with previous studies showing less social attention in ASD (40–42). While a trend for reduced attention to faces in ASD was found, the differences were small and ASD adults viewed eyes and mouths for a similar amount of time as their TD peers. However, the correlation between fixating at the faces and self-reported autism symptom severity found in this study indicates that those at the extreme end of the ASD continuum tend to fixate less at social cues, which might affect their social competence in general. This is in line with earlier findings by Norbury et al. (43), who found that less relative fixation at eyes is associated with more problems in social competence in adolescents with autism. We conclude that, even though these findings suggest that less attention to the environment and to specific social cues does not continue into adulthood for most individuals with normal IQ and autism, those individuals with higher autism symptoms severity may continue to show deviant social attention patterns while growing up. This should, however, be confirmed in a longitudinal study from childhood into adulthood.

The finding of lower arousal observed at rest and during social emotional stimuli processing in the ASD individuals in this study is in line with previous findings in both children (17, 44) and adults with ASD (24, 45), and is suggestive of fundamental abnormalities in the ANS in autism. Resting SCL influences elicitation of an orienting response to salient information in the environment and assists in the generation of action and approach within an organism. Atypical autonomic arousal could therefore play a role in preventing the individual from emotionally engaging in appropriate social behavior, even if the explicit cognitive performance (e.g., verbal labelling) remains intact (46). Mathersul et al. (24) showed that in a subgroup of ASD adults with lower arousal, resting SCL was related to poorer emotion recognition while the ASD group with high arousal performed similar to the TD group on emotion recognition. Moreover, the correlations between arousal and empathy scores showed negative correlations for both cognitive and affective empathy in the high-SCL ASD group, but only significant negative correlations with cognitive empathy in the low-SCL ASD group. This relation between arousal and processing of emotions is further supported by a study combining skin conductance and neural responses during rest by Eilam-Stock et al. (44), which reported lower SCR and a weaker correlation between arousal and frontal brain regions, important for social cognition, emotion, and attention in the ASD group, compared to a TD group. We conclude that resting state arousal as well as arousal during processing of emotional information is reduced in young adults with ASD and emphasize the importance of considering baseline arousal levels in individuals with ASD, both in future studies and in treatment responsivity.

At the same time, self-reported emotional awareness appeared to be congruently reduced in the ASD group in this study. The lower rating of emotions seems adequate, since the physiology measures suggest that these young adults with ASD were less aroused. This finding is in line with Dziobek et al. (20) and Trimmer et al. (19) in showing that adults with ASD show no difficulties in rating their own emotional reactions in response to stimuli. However, it should be taken into account that the high co-occurrence of alexithymia (the subclinical inability to identify and describe emotions in the self) in individuals with ASD (47) could have interfered with the ASD participants’ ability to identify and report on their emotions as has been found before in a comparable, but independent, sample of adults with autism (9). Future studies on emotional arousal in ASD are therefore recommended to include validated measures of alexithymic traits to explain additional variance in the outcome measures. Furthermore, lower emotional arousal was found to be related to higher autism symptom severity for all participants. This finding suggests that emotional hypo-arousal may be linked to atypical social functioning in general, but it might also implicate that those who report more problems in social responsiveness are more prone to low emotional arousal, while implicit processing of social emotional cues may not be atypical. This speculatively indicates an inadequate orientation and action response in autism. Lastly, the link between lower emotional arousal and higher autism symptom severity indicated by higher SRS scores could be associated with atypical sensory responsiveness in autism (48). Social interactions provide high sensory input, which might be challenging for autistic individuals to process and respond accordingly given their sensory atypicalities (49). Therefore, future research should consider the role of sensory experiences in explaining basal autonomic arousal and autonomic reactivity to socially loaded situations.

In this study several limitations exist. First, the current sample consists of a group of ASD individuals with above-average IQ and a moderately sized control group, both of whom included only a small proportion of female participants, which narrows the generalizability of the findings. Second, use of the SRS-A self-report for screening intelligent adults with autism, who might be extra aware and able to verbalize any problems they experience in social responsiveness, is suboptimal. Third, AOIs for eyes and mouth might have been too small in some video clips to detect group differences; however, larger AOIs could not be defined as that would result in overlap between the AOIs leading to false positive fixations (50). Also, the background of the video clips was computed by detracting faces from the whole screen, while no other distinction was made between social and non-social cues. Future studies using dynamic video clips could make a clearer distinction between social and non-social cues in the background, and are advised to use substantially large AOIs. And even though the video clips were counterbalanced, it is still possible that the order in which they were presented introduced interaction effects that may have influenced our findings. Another limitation of the current study is that participants were not excluded based on their current medication use, while medication use can have an effect on arousal. To check for potential influence of medication, *post hoc* analyses were run without participants who use medication that can have an effect on the arousal measurements, and this did not affect the outcome in terms of significance. Therefore, it was decided to keep medicated participants in the dataset to maximize representativeness of our sample for the general ASD population. Finally, the fact that ASD participants were not tested in the lab while the control participants were could have had an influence on our results as technical problems with our testing devices were more easily resolved in the lab environment than in the residential houses of the ASD participants. However, the clear benefit of maximizing inclusion by providing on-site assessments outweighed the potential disadvantage of introducing measurement error due to site differences.

In conclusion, these findings may partially help explain why young adults with ASD are less inclined to show adaptive social behavior in an emotionally loaded context. Improving emotion regulation in ASD deserves our full attention as this might help these young adults in navigating through daily social emotional situations.

## Ethics Statement

The research protocol was approved by the Medical Ethics Committee of Leiden University Medical Center. In accordance with the declaration of Helsinki, written informed consent was obtained from all participants before participation.

## Author Contributions

WS and HS devised the project, the main conceptual ideas, and proof outline. RD and EG performed the measurements under the supervision of TZ. HS was involved in planning and supervised the work. EG, RD, and TZ processed the experimental data, performed the analysis, drafted the manuscript, and designed the figures. All authors discussed the results and commented on the manuscript.

## Funding

This work was supported by the scientific board of Jados. The funding source had no involvement in the study or writing of the report.

## Conflict of Interest Statement

This work was supported by the scientific board of the independent foundation Jados. RD is employed as a tutor at Stumass and WS and HS are board members of the scientific board of Jados. The authors report no biomedical financial interests or potential conflicts of interest.
